# Interleukin-27 Protects Cardiomyocyte-Like H9c2 Cells against Metabolic Syndrome: Role of STAT3 Signaling

**DOI:** 10.1155/2015/689614

**Published:** 2015-08-03

**Authors:** Wei-Lian Phan, Yu-Tzu Huang, Ming-Chieh Ma

**Affiliations:** ^1^Department of Cardiology, Cardinal Tien Hospital, New Taipei City 23148, Taiwan; ^2^School of Medicine, Fu Jen Catholic University, New Taipei City 24205, Taiwan

## Abstract

The present results demonstrated that high glucose (G), salt (S), and cholesterol C (either alone or in combination), as mimicking extracellular changes in metabolic syndrome, damage cardiomyocyte-like H9c2 cells and reduce their viability in a time-dependent manner. However, the effects were greatest when cells were exposed to all three agents (GSC). The mRNA of glycoprotein (gp) 130 and WSX-1, both components of the interleukin (IL)-27 receptor, were present in H9c2 cells. Although mRNA expression was not affected by exogenous treatment with IL-27, the expression of gp130 mRNA (but not that of WSX-1 mRNA) was attenuated by GSC. Treatment of IL-27 to H9c2 cells increased activation of signal transducer and activator of transcription 3 (STAT3) and protected cells from GSC-induced cytochrome c release and cell damage. The protective effects of IL-27 were abrogated by the STAT3 inhibitor, stattic. The results of the present study clearly demonstrate that the STAT3 pathway triggered by anti-inflammatory IL-27 plays a role in protecting cardiomyocytes against GSC-mediated damage.

## 1. Introduction

In humans, metabolic syndrome (MS) is characterized by a combination of obesity, hyperglycemia, glucose intolerance, dyslipidemia, and hypertension [1]. The risk of heart attack is three times greater for those with MS than for those without [2]. Previous studies show that MS increases mortality in patients with acute myocardial infarction and both during and after coronary artery bypass surgery [3–5]. Thus, MS renders the myocardium intolerant to further injury, including ischemia or mechanical damage. This notion is supported by a recent study showing that MS increases apoptosis in rat cardiomyocytes after myocardial ischemia/reperfusion (IR) injury via reactive oxygen species- (ROS-) mediated increases in mitochondrial permeability [6]. Thus, it is necessary to explore the mechanism underlying the effects of MS on the myocardium with a view on developing new treatments for heart diseases associated with metabolic disorders.

Inflammation links MS and heart disease [7]. Metabolic overload triggers oxidative stress, organelle dysfunction, and cell hypertrophy, all of which generate a vicious self-amplifying cycle that leads to inflammation [1]. For example, hypertrophy of adipose tissue (which is an active endocrine organ) causes cell rupture; this releases large amounts of cytokines, such as interleukin- (IL-) 6, tumor necrosis factor-*α*, and adiponectin, all of which cause inflammation [1, 7, 8]. IL-6 has been identified as an inflammatory marker in MS; however, animal studies show that this cytokine has protective effects in patients with unstable angina and ameliorates IR injury [9–11]. Interestingly, these protective effects are mostly related to the activation of signal transducer and activator of transcription 3 (STAT3). IL-6 family cytokines activate glycoprotein (gp) 130 and phosphorylate STAT3, which is then translocated to the cell nucleus where it activates genes that control cell proliferation, differentiation, and survival [12–14]. Increased STAT activity upregulates the expression of several cardioprotective genes, including B-cell lymphoma-extra large, which prevents myocardial apoptosis and inhibits vascular endothelial growth factor [15, 16].

IL-27 belongs to the IL-6 family. It is a heterodimeric cytokine comprising Epstein-Barr virus-induced gene-3 and p28 (IL-27p28) [17]. Its receptor is also a heterodimer, comprising gp130 and WSX-1 [18]. IL-27 is primarily produced by dendritic cells (DCs) and regulates the proliferation and activation of other immune cells [17, 19]. In the rat heart, DCs reside close to endocardial blood vessels and align parallel to the myocardium with their processes interdigitating between the cardiomyocytes [20]. Studies in the IR rat model show that DCs accumulate within the zones bordering infarcted tissue 7 days after myocardial infarction [21]. Moreover, circulating levels of IL-27 are elevated in patients with coronary artery disease [22]. Taken together, these results indicate that IL-27 released by DCs located around peri-infarcted tissues may aid myocardial recovery from postischemic insult.

Our previously results show that IL-27 attenuates IR-induced cardiac injury in isolated rat hearts and protects H9c2 cells against severe hypoxia (2% oxygen) induced cell death [23]. These protective effects were blocked by the small-molecule STAT3 inhibitor, stattic. The aim of the present study was therefore to examine whether IL-27 activates the STAT3 pathway to protect H9c2 cells against phenotypic changes associated with MS. Because hyperglycemia, hypertension, and dyslipidemia are the most important symptoms in MS and because of lack of definition of MS at cellular level, we applied high glucose (G), high NaCl (S), and/or cholesterol (C) treatment to mimic the phenotype and/or extracellular changes associated with MS. We first compared the effects of G, S, and C, either individually or in combination, on cell viability and used RT-PCT to examine their effects on IL-27 receptor expression in treated H9c2 cells. We then further examined the effect of IL-27 by exposing cells to the cytokine in the presence and absence of stattic. Quantitative changes in STAT3 activity under different treatment conditions were measured using enzyme-linked immunosorbent assay (ELISA) kits.

## 2. Materials and Methods

### 2.1. Chemicals

IL-27 and stattic (6-nitro-1,1-dioxide-benzo[b]thiophene) were obtained from R&D Systems (Minneapolis, MN) and Merck (Darmstadt, Germany), respectively, and were prepared in sterile water. S, G, and water-soluble C, 3-(4,5-cimethylthiazol-2-yl)-2,5-diphenyl tetrazolium bromide (MTT), and dimethyl sulfoxide (DMSO) were purchased from Sigma-Aldrich (St. Louis, MO).

### 2.2. Culture of Cardiomyocyte-Like H9c2 Cells

The cardiomyoblast cell line, H9c2, was purchased from the Bioresource Collection and Research Center (Hsinchu, Taiwan). The cell line was originally derived from the American Type Culture Collection line, CRL-1446. All culture media and supplements were purchased from Thermo Scientific HyClone (South Logan, UT). H9c2 cells were routinely cultured at 37°C/5% CO_2_ in Dulbecco's modified Eagle's medium containing 10% fetal bovine serum (FBS), 1% L-glutamine, penicillin (100 U mL^−1^), streptomycin (100 mg mL^−1^), and amphotericin B (0.25 *μ*g mL^−1^), as described previously [24]. The concentrations of S, G, and C in the culture medium were 155 mM, 5 mM, and zero, respectively. Cells were subcultured when they reached 80% confluence (approximately every 2-3 days). Two days before each experiment, cells were subcultured in 48 well plates (1.5 × 10^4^/well) and grown in culture medium containing 5% FBS to avoid overstimulation of STAT3. Pilot results showed that cells adapted well under these conditions, with no evident change in lactate dehydrogenase (LDH) release or MTT staining after 48 h of incubation.

On the day of the experiment, cells were pretreated with sterile water (vehicle), IL-27 (150 ng mL^−1^), or stattic (10 *μ*M) for 4 h. Dosing was based on cell viability. Next, the cells were exposed to G (25 mM), S (250 mM), and C (300 *μ*g mL^−1^) either alone or in different combinations (GS, SC, GC, or GSC) for 4, 8, 24, or 48 h to mimic MS.

### 2.3. Quantitative Real-Time PCR

RNA was extracted using a commercial kit (RareRNA, Bio-East Technology, Taipei, Taiwan) as previously described [25] and cDNA synthesized at 42°C for 45 min (reaction mixture: 2 *μ*g RNA, 5 *μ*g of poly(dT)15 oligonucleotide primer (Life Technologies), and 200 units of reverse transcriptase (Moloney murine leukemia virus; Promega, Madison, WI)). Quantitative RT-PCR was performed in an ABI StepOne Plus system (Applied Biosystems, Foster City, CA). PCR was performed using 100 ng of cDNA and 30 *μ*mol of primers (total reaction volume, 20 *μ*L) using the SYBR Green PCR master mix kit according to the manufacturer's instructions (Applied Biosystems). The following primers were used for PCR: gp130, 5′-ATA CCT TAA ACA AGC TCC ACC TTC-3′ (forward) and 5′-AGT TTC ATT TCC AAT GAT GGT TCT-3′ (reverse); WSX-1, 5′-GAA ACC CAA ATG AAG CCA AA-3′ (forward) and 5′-GCC TCC TGA CAT CTT CGG TA-3′ (reverse); glyceraldehyde 3-phosphate dehydrogenase (GAPDH), 5′-TTA GCA CCC CTG GCC AAG G-3′ (forward) and 5′-CTT ACT CCT TGG AGG CCA TG-3′ (reverse). The cycling conditions were as follows: 95°C for 20 s, followed by 40 cycles of 95°C for 1 s and 60°C for 20 s. Melting curve analysis was performed at the end of each PCR experiment. All reactions were run in duplicate. The ΔCt (threshold cycle) was calculated by subtracting the raw Ct values for the house-keeping gene (GAPDH) from the raw Ct values for the target gene, thereby providing information about relative changes in gene expression. Changes in target gene expression were calculated as 2^−ΔCt^ and expressed as the fold change relative to that in control cells.

### 2.4. Cell Cytotoxicity/Viability Assays

To assess cytotoxicity, LDH levels were measured in cell culture medium (20 *μ*L) at different time points using a commercial kit (Roche Applied Science, Indianapolis, IN), as described previously [24]. The advantage of LDH assay is that it allows cell viability to be continuously monitored. An MTT assay was used to measure cell viability. Briefly, cells were cultured for 48 h in the presence or absence of the different agents. The medium was then removed and the cells were washed three times with phosphate-buffered saline (PBS, pH 7.4). MTT (5 mg mL^−1^ in PBS) was then added to cells for 4 h at 37°C. MTT formazan crystals were then dissolved in DMSO and the optical density (O.D.) measured in an ELISA plate reader (Amersham-Pharmacia Biotech, Piscataway, NJ) at 570 nm. Cell viability (%) was calculated using the following formula: (viable cells) % = (O.D. of treated sample/O.D. of control sample) × 100.

### 2.5. Measurement of Phosphorylated STAT3 and Total STAT3

Commercial ELISA kits (eBioscience, San Diego, CA) were used to measure the levels of phosphorylated and total STAT3, according to the manufacturer's instructions. Briefly, cells were lysed in lysis buffer supplied with the kit at room temperature for 20 min. The protein concentration in each sample was then measured using a colorimetric assay kit (Bio-Rad, Hercules, CA) to confirm that the amount of protein was sufficient for further tests. The lysed sample and the positive control (supplied with kit) were then transferred to the coating plate and mixed with an equivalent volume of the antibody cocktail supplied with the kit. The mixture was then incubated in the dark for 2 h. After washing, the detection reagent was added for 30 min. Finally, stop solution was added to each well and the colorimetric reaction was measured at 450 nm. STAT3 activity was expressed as a ratio of the amount of phosphorylated to total STAT3 after normalization against the total protein concentration.

### 2.6. Western Blot Analysis

The expression of cytochrome c (an indicator of apoptosis) and actin was examined by immunoblot analysis, as described previously [24, 25]. Primary antibodies against cytochrome c (cyto c) and actin were obtained from Santa Cruz Biotechnology (1 : 2000; Santa Cruz, CA). Briefly, equal amounts of cytosolic protein were separated in denaturing SDS polyacrylamide gels and electrophoretically transferred to polyvinylidene difluoride membranes (Amersham, Little Chalfont, UK). The membranes were then incubated with the appropriate primary antibodies overnight at 4°C. After washing, the membranes were incubated for 1 h at room temperature with the corresponding horseradish peroxidase-conjugated secondary antibodies (1 : 200; Vector Laboratories, Burlingame, CA). Bound antibodies were visualized using an enhanced chemiluminescence kit (Amersham-Pharmacia Biotech) and Kodak film. Band density was measured semiquantitatively using an image analytical system (Diagnostic Instruments, Sterling Heights, MI). The amount of each protein was expressed relative to the amount of actin.

### 2.7. Statistical Analysis

All data are expressed as the mean ± standard error of the mean (S.E.M.). Statistical analysis was performed using analysis of variance or analysis of variance followed by the Newman-Keuls method for multiple comparisons between groups. A *P* value < 0.05 was considered significant.

## 3. Results

### 3.1. High Glucose, High Salt, and Cholesterol Induce Cell Injury

**Figure 1:**
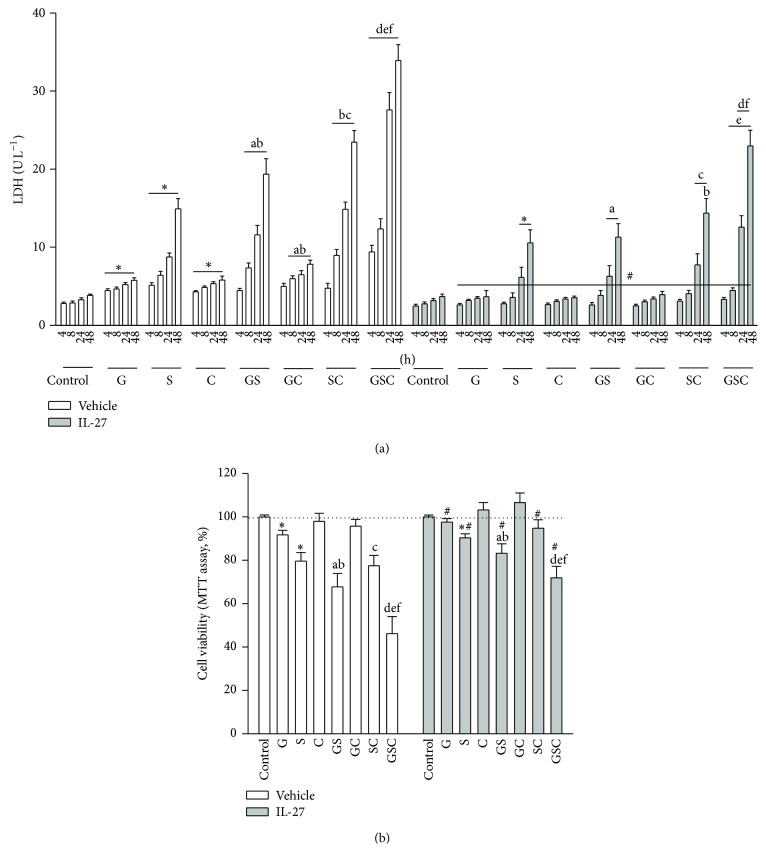
Cytotoxic effects of high glucose, high sodium, or high cholesterol on the viability of H9c2 cells. (a) Cells were treated with glucose (25 mM, G), NaCl (250 mM, S), or cholesterol (300 *μ*g mL^−1^, C), either alone or in different combinations, for 4, 8, 24, and 48 h. Cells were treated with IL-27 4 h before treatment with G, S, or C. Lactate dehydrogenase (LDH) release was measured as marker of cell injury. (b) Cells were cultured for 48 h in the presence of G, S, or C (either alone or in different combinations) and cell viability was monitored in an MTT assay. *N* = 6 of experiments performed at each time point. ^*^ *P* < 0.05 versus the control group; ^a^ *P* < 0.05 versus G; ^b^ *P* < 0.05 versus S; ^c^ *P* < 0.05 versus C; ^d^ *P* < 0.05 versus GS; ^e^ *P* < 0.05 versus GC; ^f^ *P* < 0.05 versus the SC; and ^#^ *P* < 0.05 versus the vehicle-treated control at the same time-point.

Treatment of cells with G, S, or C alone led to a time-dependent increase in LDH release from H9c2 cells (Figure 1(a), open bars). In addition, G or S alone led to a reduction in cell viability after 48 h of treatment (Figure 1(b), open bars). Cell damage was more severe when cells were exposed to a combination of all three agents (GSC), particularly after 24 h and 48 h. A significant reduction in cell viability was noted when cells were treated with S plus G or C, but not when cells were treated with G plus C in the absence of S. However, when cells were treated with GSC, cell viability was reduced to ~58% of that in control cells.

Prior treatment with IL-27 markedly attenuated cell damage and death caused by G, S, or C alone (Figures 1(a) and 1(b), gray bars). However, significant changes were only observed after 24 h and 48 h of treatment with S. The synergistic effects of GSC on cell viability were abrogated by pretreatment with IL-27.

### 3.2. IL-27 Receptor Expression

**Figure 2:**
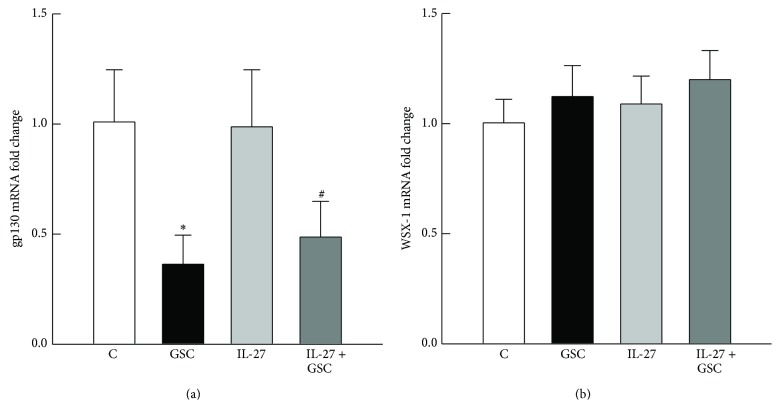
Expression of IL-27 receptor mRNA. The expression of gp130 (a) and WSX-1 (b) mRNA in control cells and cells treated with GSC and/or IL-27 was examined by RT-PCR. *N* = 6 of experiments performed in each group. ^*^ *P* < 0.05 versus the control (C) group; ^#^ *P* < 0.05 versus the IL-27 group.

The mRNA for gp130 and WSX-1 was detected by RT-PCR, indicating that H9c2 cells express IL-27 receptors. However, the expression of gp130 mRNA was markedly attenuated in cells treated with GSC (0.37 ± 0.11 versus 1.0 ± 0.13 in controls, *P* < 0.05, Figure 2(a)). IL-27 had no effect on gp130 mRNA expression in either the presence or absence of GSC (0.45 ± 0.12 in the IL-27 + GSC group). Neither GSC nor IL-27 affected the expression of WSX-1 mRNA (Figure 2(b)).

### 3.3. IL-27 Increases STAT3 Activity and Inhibits the Release of Cytochrome c

**Figure 3:**
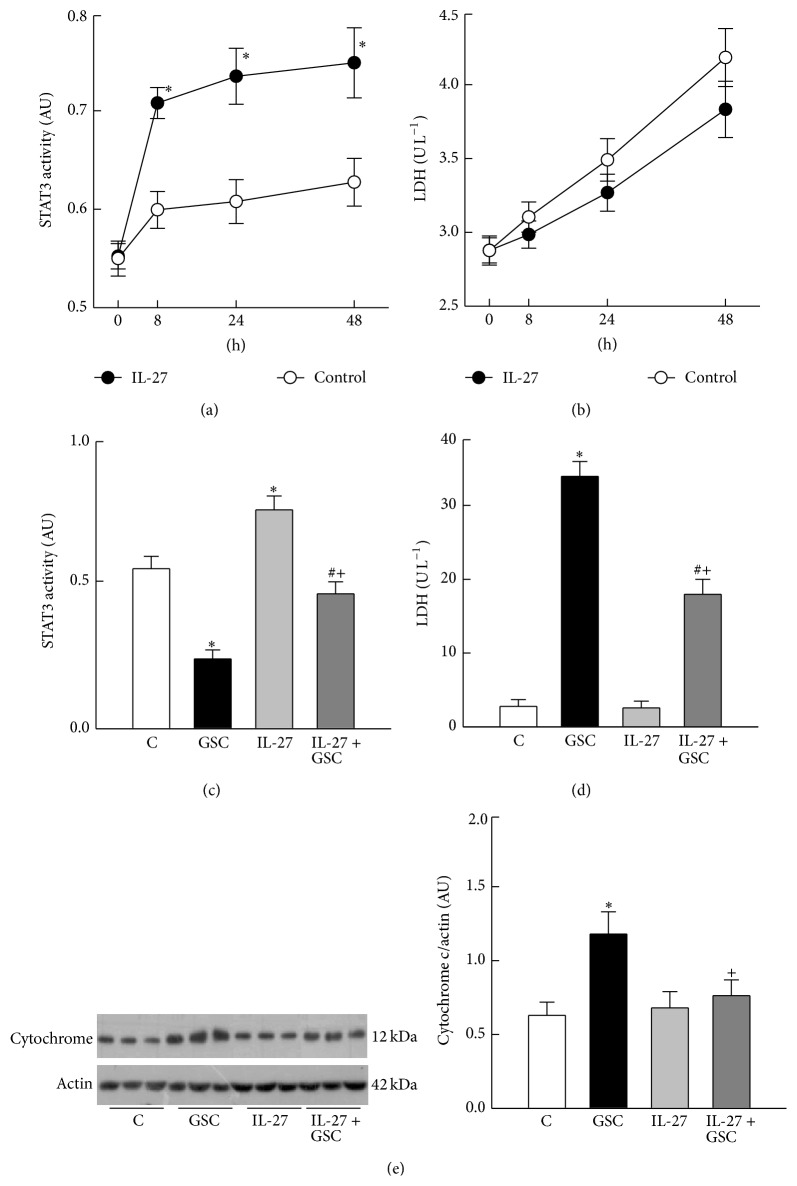
Effects of IL-27 on STAT3 activity and cytochrome c release from H9c2 cells. (a) IL-27 increased the activity of STAT3 in a time-dependent manner compared with that in control (vehicle-treated) cells. (b) Lactate dehydrogenase (LDH) release by control cells indicates spontaneous turnover during cell growth. Note that IL-27 had no effect on LDH release. (c) Changes in STAT3 activity after 48 h of treatment with GSC, IL-27, or GSC + IL-27. Note that IL-27 partially reversed the glucose, sodium, and cholesterol (GSC) induced reduction in STAT3 activity. (d) Effects of GSC and IL-27 on LDH release. Note that the GSC-mediated increase in LDH release was attenuated by IL-27. (e) Apoptosis was evaluated by assessing the cytosolic levels of cytochrome c (Cyto c). Western blots showing the expression of cytochrome c in three representative cultures. Equal amounts of protein (20 *μ*g per lane) were loaded. The bar graph shows changes in cytochrome c expression (as measured by densitometry) after normalization against actin. *N* = 6 of experiments performed at each time-point or in each group. AU, arbitrary units. ^*^ *P* < 0.05 versus the zero time-point or the control (C) group; ^#^ *P* < 0.05 versus the IL-27 group; ^+^ *P* < 0.05 versus the GSC group.

Binding of IL-27 to gp130 triggers STAT3 activation, which then transduces downstream signals to elicit cellular responses [17]. Therefore, we next examined whether IL-27 affects STAT3 activity in H9c2 cells. Prior to treatment with IL-27, cells showed similar levels of STAT3 activity (Figure 3(a)). STAT3 activity was significantly higher in cells exposed to IL-27 alone for 8, 24, and 48 h than in vehicle-treated cells. However, IL-27 had no significant effect on LDH release (Figure 3(b)). Interestingly, cells treated with GSC for 48 h showed lower STAT3 activity (0.24 ± 0.03 versus 0.54 ± 0.04 in controls, *P* < 0.05, Figure 3(c)) and increased LDH release (33.9 ± 2.1 versus 2.8 ± 0.9 U L^−1^ in controls, *P* < 0.05, Figure 3(d)). Pretreatment with IL-27 prevented the GSC-induced reduction in STAT3 activity (0.45 ± 0.04, *P* < 0.05 versus GSC group) and cell injury (17.9 ± 2.0 U L^−1^, *P* < 0.05 versus GSC group). GSC treatment significantly increased the expression of cytochrome c in the cytosolic fraction (1.18 ± 0.15 versus 0.62 ± 0.09 in controls, *P* < 0.05, Figure 3(e)), suggesting that GSC induces cytochrome c release. This GSC-induced increase in cytochrome c was abrogated by IL-27 (0.76 ± 0.11, *P* < 0.05 versus GSC group).

### 3.4. STAT3 Inhibition Abrogates IL-27-Mediated Myocardial Protection

**Figure 4:**
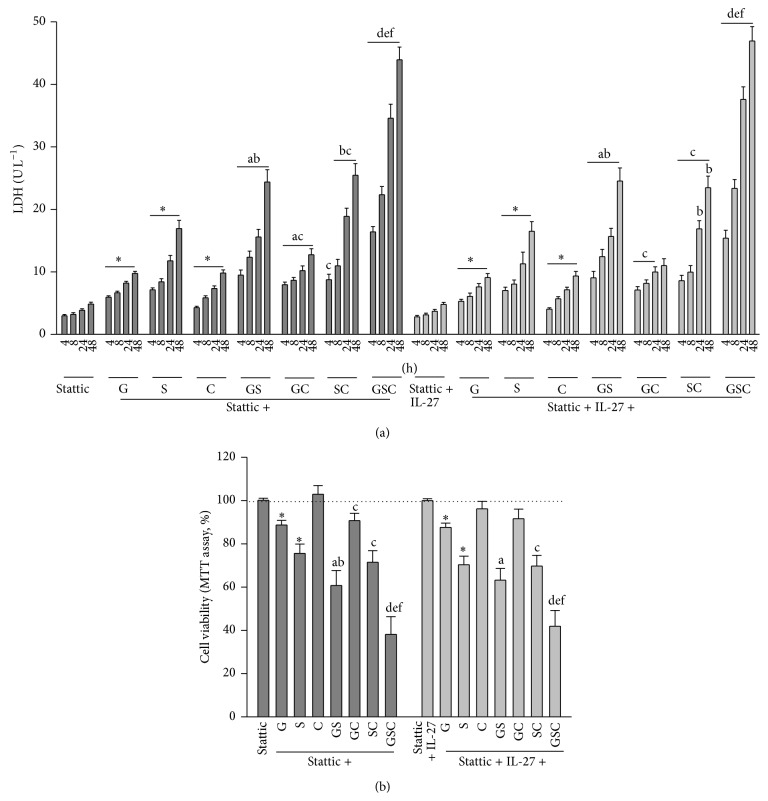
Effect of STAT3 inhibition on cell viability. (a) Cells were treated with stattic (dark gray bars) or stattic plus IL-27 (light gray bars) 4 h prior to treatment with glucose (25 mM, G), NaCl (250 mM, S), or cholesterol (300 *μ*g mL^−1^, C), either alone or in different combinations, for 4, 8, 24, and 48 h. Lactate dehydrogenase (LDH) was determined as a marker of cell injury. (b) Cells were cultured for 48 h in the presence of the indicated agents and viability was measured in an MTT assay. *N* = 6 of experiments performed in each time-point of group. ^*^ *P* < 0.05 versus the corresponding control groups (stattic alone or stattic + IL-27); ^a^ *P* < 0.05 versus G; ^b^ *P* < 0.05 versus S; ^c^ *P* < 0.05 versus C; ^d^ *P* < 0.05 versus GS; ^e^ *P* < 0.05 versus GC; ^f^ *P* < 0.05 versus SC.

The above results clearly indicate that increased STAT3 activity plays a role in IL-27-mediated myocardial protection. Therefore, we next examined the effect of a selective STAT3 inhibitor, stattic, on the effects of IL-27. Cells were treated with stattic alone (dark gray bars) or stattic + IL-27 (light gray bars) 4 h prior to treatment with G, S, or C, either alone or in different combinations. Stattic alone had no effect on LDH release by control cells (Figure 4(a)). However, there was no difference in LDH release or cell viability after cotreatment with G, S, and C alone, or in different combinations, and stattic or stattic + IL-27 (Figures 4(a) and 4(b)).

### 3.5. Stattic Reverses the Effects of IL-27

**Figure 5:**
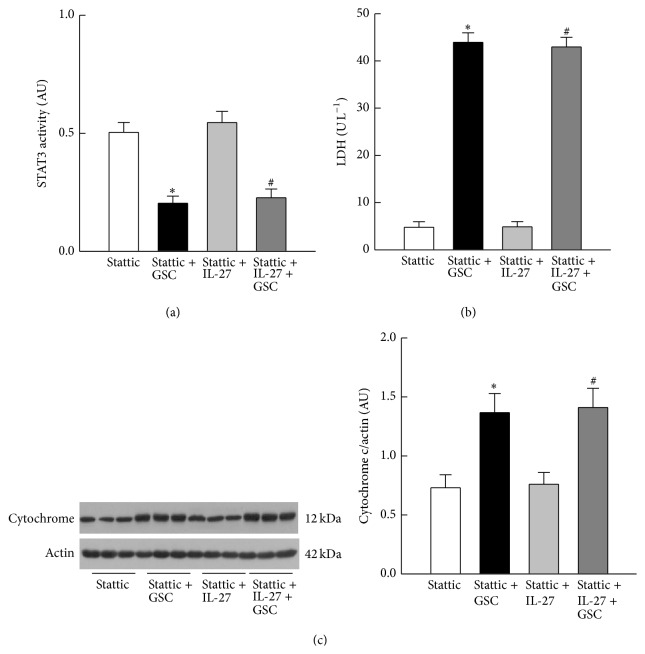
Effect of stattic on STAT3 activity and cytochrome c release. (a) Changes in STAT3 activity after 48 h of treatment with stattic, stattic + GSC, stattic + IL-27, or stattic + IL-27 + GSC. Note that stattic enhanced the high glucose, sodium, and cholesterol (GSC) mediated reduction in STAT3 activity and abrogated the STAT3-enhancing effects of IL-27. (b) Stattic increased LDH release in GSC-treated cells in the presence or absence of IL-27. (c) Apoptosis was evaluated by measuring cytochrome c (Cyto c) release. Western blots showing the expression of cytochrome c in three representative cell cultures. Equal amounts of protein (20 *μ*g per lane) were loaded. The bar graph shows changes in the expression of cytochrome c in treated cells (after being normalized against actin). *N* = 6 of experiments performed in each group. AU, arbitrary units. ^*^ *P* < 0.05 versus the stattic group; ^#^ *P* < 0.05 versus the stattic + IL-27 group.

We next examined whether stattic abrogated the protective effects of IL-27 in H9c2 cells. Compared to Figure 3(c), stattic had no effect on reduction in STAT3 activity in cells treated with GSC but led to a reduction in STAT3 activity in cells treated with IL-27 alone (Figure 5(a)). However, it did reduce STAT3 activity in cells treated with both IL-27 and GSC (Figure 5(a)). Cotreatment with stattic or with stattic plus IL-27 increased the levels of GSC-mediated cell damage (Figure 5(b)) more than those in Figure 3(d). However, stattic did not affect the GSC-induced increase in cytochrome c levels in the cytoplasm (Figure 5(c)). A similar increase in cytochrome c expression was observed in GSC cells cotreated with stattic plus IL-27. These results clearly indicate that IL-27-mediated cardioprotection against GSC-induced cell damage and apoptosis is dependent upon STAT3 activation.

## 4. Discussion

The results of the present study show that the IL-27/STAT3 pathway plays a role in protecting cardiomyocytes against GSC-mediated damage. First, we showed that G, S, and C (either alone or in combination) damage H9c2 cells and reduce their viability; however, the effects were greatest when cells were exposed to all three agents (GSC). Second, we found that H9c2 cells expressed mRNA for gp130 and WSX-1, both components of the IL-27 receptor. Although mRNA expression was not affected by exogenous treatment with IL-27, the expression of gp130 mRNA (but not that of WSX-1 mRNA) was attenuated by GSC. Third, binding of IL-27 to its receptor on H9c cells increased activation of STAT3 and protected cells from GSC-induced cytochrome c release and cell damage. Finally, the protective effects of IL-27 were abrogated by the STAT3 inhibitor, stattic.

Treatment with high glucose, high sodium, fatty acids, or cholesterol derivatives induce apoptosis in H9c2 cells and adult rat cardiomyocytes [26–28]. Here, we showed that GSC treatment caused cell damage and apoptosis in H9c2 cardiomyoblasts. High glucose, high sodium, and/or fatty acid levels can injure cardiomyocytes via several mechanisms, including oxidative stress, activation of nuclear factor-*κ*B, increased release of inflammatory cytokines (e.g., IL-1*β*, IL-6, and TNF-*α*), and apoptosis [26–28]. Here, we identified a novel mechanism of cardiac cell injury induced by GSC-mediated suppression of STAT3 activity.

Binding of growth factors or cytokines to gp130 activates Janus kinase, which in turn recruits and activates STAT3, which is then translocated to the nucleus where it transactivates its target genes [29]. We speculated that GSC impairs the survival-promoting effects of growth factors or cytokines on cardiac cells, thereby inducing cell death. This notion is supported by our observations of reduced STAT3 activity and defects in gp130 mRNA expression in GSC-treated H9c2 cells. Gp130 is utilized by several growth factors and cytokines; therefore, reduced gp130 expression may affect cell fate. We found that although IL-27 had no effect on the GSC-mediated reduction in gp130 mRNA expression, it did induce a significant increase in STAT3 activity. This may be due to the enhanced function of residual gp130 expressed by GSC-treated cells, which triggers increased STAT3 activation. However, because cardiomyocytes release cytokines upon cardiac injury, we cannot rule out the possibility that impaired release of such growth factors adversely affects endogenous repair mechanisms [30]. Interestingly, neither GSC nor IL-27 affected the expression of WSX-1 mRNA. The association between WSX-1 and gp130 inhibits inflammation [31]. Genetic defects in WSX-1 make animals more susceptible to lethal inflammation induced by parasitic infection [31]. Therefore, it may be that the WSX-1 expression observed in GSC-treated H9c2 protects the cells by allowing STAT3 activation. Further studies are needed to examine whether WSX-1 blockade exacerbates GSC-mediated cardiomyocyte injury. Moreover, measurements of IL-27 receptor mRNA probably do not reflect the protein expression on the surface of cells. Further studies are required to examine the protein levels of gp130 and WSX-1 after MS and/or IL-27 treatment, and this is an experimental limitation of the study.

Though the present study did not explore the downstream effects mediated by STAT3, previous studies show that STAT3 phosphorylation targets both nucleus and mitochondria [14]. Key residues within the STAT3 protein, that is, tyr705 and ser727, must be phosphorylated for maximum transcriptional activity in the nucleus [14]. Phosphorylation at these sites is required for STAT3 to play its roles in cell survival, proliferation, and differentiation and in preventing apoptosis [32]. The commercial kit used in the present study was able to detect STAT3 phosphorylation at both these crucial sites. About 10% of cytosolic STAT3 resides within the mitochondria where it binds to complex I or II in the electron transport chain to drive ATP synthesis [14, 33]. These interactions, however, require that STAT3 be phosphorylated only on ser727, not tyr705 [14, 33]. Moreover, a previous study showed that the mitochondrial distribution of STAT3 plays an important role in inhibiting mitochondrial permeability transition pore (MPTP) opening [34], which allows the release of cytochrome c and induces myocardial apoptosis and necrosis. The results of the present study are consistent with this, as STAT3-mediated protection of H9c2 cells by IL-27 was associated with reduced cytochrome c release and was abrogated by stattic. As the MPTP opens in response to high concentrations of ROS and calcium, we speculate that IL-27-mediated STAT3 activation may improve antioxidant defense and maintain calcium homeostasis. Indeed, transgenic mouse hearts expressing constitutively active STAT3 show upregulated expression of the free radical scavenger, metallothionein [35]. Further studies should explore the role of STAT3 in protecting against calcium overload during heart failure. Calcium overload results from a lack of ATP in failed cardiomyocytes; however, STAT3 plays a unique role in regulating ATP synthesis when targeted to mitochondria.

STAT3 is an essential component of the Survivor Activating Factor Enhancement (SAFE) pathway and, as such, it can reduce cardiomyocyte death at the time of reperfusion after ischemic insult [14]. Thus, several cardioprotective strategies and agents that activate STAT3 can successfully rescue injured cardiomyocytes via the SAFE pathway; such strategies and agents include ischemic conditioning, cardiotrophin-1, urocortin, opioids, insulin, leptin, resveratrol, melatonin, and erythropoietin [36–43]. Though the present study did not examine the effects of IR injury, our previous data show that IL-27-induced STAT3 activation is important for attenuating IR and hypoxic injury to rat hearts or H9c2 cells, respectively [23]. Here, we identified a novel role for IL-27 in cardioprotection via activation of STAT3 and the SAFE pathway. Studies show that the diet-induced obesity and insulin resistance associated with MS reduce myocardial resistance to IR injury [44]. Obesogenic hearts express low levels of molecules involved in the reperfusion injury salvage kinase (RISK) pathway, including Akt and glycogen synthase kinase 3*β* [44]. Moreover, previous findings in chick embryonic hearts reveal that protein kinases within the RISK pathway are activated downstream of STAT3 [45]. Therefore, further studies should examine whether the RISK pathway participates in IL-27/STAT3 signaling to protect against MS-mediated cardiac injury. Furthermore, a number of mechanisms, such as suppressors of cytokine signaling (SOCS) and the protein inhibitors of activated STATs (PIAS), also inhibit STAT activity as part of a negative feedback control mechanism [12, 14]. It is not clear whether these negative regulatory mechanisms are present in cardiomyocytes; if so, then further studies should examine whether their functions are affected by GSC or IL-27. Such studies may provide a more complete picture of the effects of IL-27 with respect to cardioprotection.
